# Camel Milk as a Potential Therapy as an Antioxidant in Autism Spectrum Disorder (ASD)

**DOI:** 10.1155/2013/602834

**Published:** 2013-08-29

**Authors:** Laila Y. AL-Ayadhi, Nadra Elyass Elamin

**Affiliations:** ^1^Department of Physiology, Faculty of Medicine, King Saud University, P.O. Box 2925, Riyadh 11461, Saudi Arabia; ^2^Autism Research and Treatment Center, Shaik AL-Amodi Autism Research Chair, Faculty of Medicine, King Saud University, P.O. Box 2925, Riyadh 11461, Saudi Arabia

## Abstract

Extensive studies have demonstrated that oxidative stress plays a vital role in the pathology of several neurological diseases, including autism spectrum disorder (ASD); those studies proposed that GSH and antioxidant enzymes have a pathophysiological role in autism. Furthermore, camel milk has emerged to have potential therapeutic effects in autism. The aim of the current study was to evaluate the effect of camel milk consumption on oxidative stress biomarkers in autistic children, by measuring the plasma levels of glutathione, superoxide dismutase, and myeloperoxidase before and 2 weeks after camel milk consumption, using the ELISA technique. All measured parameters exhibited significant increase after camel milk consumption (*P* < 0.5). These findings suggest that camel milk could play an important role in decreasing oxidative stress by alteration of antioxidant enzymes and nonenzymatic antioxidant molecules levels, as well as the improvement of autistic behaviour as demonstrated by the improved Childhood Autism Rating Scale (CARS).

## 1. Introduction

Autism spectrum disorder (ASD) is a severe neurodevelopment disorder with onset prior to 3 years of age [1, 2]. It is characterized by impairments in social orientation, communication, and repetitive behaviors [3, 4]. In addition to behavioural impairment, ASD is associated with high prevalence of autoimmune disease [5, 6], gastrointestinal disease and dysbiosis [7], and mental retardation [8].

The prevalence of autism has increased over the last several decades. The incidence of ASD in United States increased in 2008 to 1 in 88 children [9]. Prevalence of autism spectrum disorders in Saudi Arabia is estimated to be 6 : 1000 [10]. Increased prevalence has great effects on public health implications and has stimulated intense research into potential etiologic factors.

Although the aetiology and pathology is poorly understood, different factors have been suggested to affect autism, for example, immune factors, environmental, neurochemical, and genetic factors [3, 10, 11], oxidative stress [10–13].

Extensive studies have demonstrated that oxidative stress plays a vital role in the pathology of several neurological diseases such as Alzheimer's disease [14], Down syndrome [15], Parkinson's disease [16], schizophrenia [17], bipolar disorder [18], and autism [10, 14]. 

Oxidative stress occurs when reactive oxygen species (ROS) levels exceed the antioxidant capacity of a cell. It acts as a mediator in brain injury, strokes, and neurodegenerative diseases [19–21]; thus, the control of ROS production is necessary for physiologic cell function. The ROS within the cells are neutralized by antioxidant defence mechanisms, including superoxide dismutase (SOD), catalase, and glutathione peroxidise (GSH-Px) enzymes. The increased production of ROS both centrally (in the brain) and peripherally (in the plasma) may result in the reduction of brain cell number leading to autism pathology and apoptosis [14, 22]. 

Several studies have suggested the contribution of oxidative stress to the development of autism. These studies demonstrated the alteration of antioxidant enzymes like GSH-Px, MPO, and SOD, lipid peroxidation, antioxidant proteins as ceruloplasmin and transferrin, and detoxifying metabolites like GSH, as well as antioxidant nutrient vitamins and minerals [10, 11, 13, 23–26]. 

Camel milk has emerged to have potential therapeutic effects in many diseases such as food allergy, diabetes mellitus [27, 28], hepatitis B [29], autism [30], and other autoimmune diseases [31]. It has a unique composition that differs from other ruminants' milk. It contains lower fat, cholesterol, and lactose than cow milk, higher minerals (calcium, iron, magnesium, copper, zinc, and potassium) and vitamins A, B2, E, and C compared to cow milk [32, 33], and it contains no beta lactoglobulin and beta casein which are the main causative of allergy in cow's milk [34]. Furthermore, camel milk contains various protective proteins, mainly enzymes which exert antibacterial, antiviral, and immunological properties [35, 36]; these include immunoglobulins, lysozymes, lactoferrin, lactoperoxidase, N-acetyl-§-glucosaminidase (NAGase), and peptidoglycan recognition protein (PGRP) [34], which are crucial in preventing food allergy and rehabilitating the immune system [31]. Camel milk proved its potential effect in the treatment of food allergies, due to its inflammation-inhibiting proteins, and hypoallergenic properties, in addition to its smaller size nanobodies, which are different than those found in human. Camel milk nanobodies, as a single domain, show many promising and therapeutic potencies in infection and immunity [37]. 

The aim of the current study was to evaluate the effect of camel milk consumption on oxidative stress biomarkers in autistic children, by measuring the plasma levels of glutathione, superoxide dismutase, and myeloperoxidase.

## 2. Materials and Methods

### 2.1. Subjects

The present study included 60 subjects with ASD, especially those with known allergies or food intolerances, aged 2–12 years. Clinical diagnosis was based on the criteria for autistic disorder as defined in the Diagnostic and Statistical Manual of Mental Disorders, Fourth Edition, Text Revision (DSM-IV) [2]. Subjects were recruited from the Autism Research and Treatment Center, Faculty of Medicine, King Saud University. 

The study protocol received the ethical approval from the Institutional Review Board of Faculty of Medicine, King Saud University. A written informed consent was obtained from all parents/guardians before being enrolled in the study.

### 2.2. Study Design

The study was a double-blinded, randomized clinical trial (RCT). The participants were randomly divided into three groups: Group I (*n* = 24) received raw camel milk; Group II (*n* = 25) received boiled camel milk; and Group III (*n* = 11) received cow milk as a placebo. All groups received the same instructions, volume of milk, and containers to preserve the blinding of the study.

Parents were instructed to include the average of 500 mL of camel milk in their children's regular daily diet for a period of 2 weeks. Parents were asked to continue with the children's daily routines. They were not allowed to add or remove any interventions such as diet plans, supplements, or pharmacotherapies throughout the study period. Group I was also instructed to drink cold milk, beginning with small quantities that increase gradually, until 500 mL per day was consumed to avoid any risk of diarrhoea.

### 2.3. Childhood Autism Rating Scale (CARS)

The Childhood Autism Rating Scale (CARS) was administered as a measure of symptom severity [11]. The Wing Subgroups Questionnaire (WSQ) [12] is a questionnaire with 13 behavioral domains (e.g., communication, social approach, play, imitation, motor behavior, and resistance to change) on which parents rate their child's behavior. A summary score is calculated for each subtype (i.e., aloof, passive, and active but odd), and the highest summary score is considered to indicate the subtype.

### 2.4. Blood Sampling

After overnight fast, ten mL blood samples were collected in EDTA tubes from autistic children before and 2 weeks after camel milk consumption. Centrifugation was done; plasma and red blood cells were obtained and deep frozen (at −80°C) until further analysis.

### 2.5. Methods

#### 2.5.1. Measurement of Glutathione

This was done by using commercially available ELISA kit (Wuhan Eiaab Science Inc., China) specific for measurement of plasma glutathione levels according to the manufacturer's instructions. Briefly, the microtiter plate has been precoated with an antibody specific to GSH. Standards and samples were pipetted into the wells with a biotin-conjugated polyclonal antibody specific for GSH. Next, avidin conjugated to horseradish peroxidase (HRP) was added and incubated. A substrate solution was added and colour developed in proportion to the amount of GSH. The colour development was stopped, and the intensity of the colour was measured.

#### 2.5.2. Measurement of Superoxide Dismutase

This assay employs the quantitative sandwich enzyme immunoassay technique for the assessment of human superoxide dismutase in plasma (Wuhan Eiaab Science Inc., China). A monoclonal antibody specific for SOD has been precoated onto a microplate. Standards and samples were pipetted into the wells, followed by addition of a second antibody specific for SOD. Then, a substrate solution was added to the wells and colour developed in proportion to the amount of SOD bound in the initial step. The colour development was stopped, and the intensity of the color was measured.

#### 2.5.3. Measurement of Myeloperoxidase

Plasma myeloperoxidase level was measured using double antibody sandwich ELISA (GenWay biotech, USA) according to the manufacturer's instructions. This method is based upon formation of enzyme-labeled antibodies complex followed by addition of chromogenic substrate to develop a color that is proportionate to the myeloperoxidase concentration.

### 2.6. Statistical Analysis

The data were analyzed and presented as mean ± SEM (standard error of the mean). Statistical differences in each measurement before and 2 weeks after milk therapy were determined with *P* values, and *P* < 0.5 was considered significant. The receiver operating characteristics (ROC) curve as a fundamental tool for biomarkers evaluation was performed using the same computer program. In a ROC curve, the true positive rate (sensitivity) is plotted in function of the false positive rate (100-specificity) for different cut-off points of a parameter. Each point on the ROC curve represents a sensitivity/specificity pair corresponding to a particular decision threshold. The area under the ROC curve is a measure of how well a parameter can distinguish between camel-milk-treated and untreated autistic subjects.

## 3. Results

The present study was performed to study the effect of camel milk consumption on oxidative stress on ASD subjects by measuring the plasma levels of glutathione, superoxide dismutase, and myeloperoxidase.

**Figure 1:**
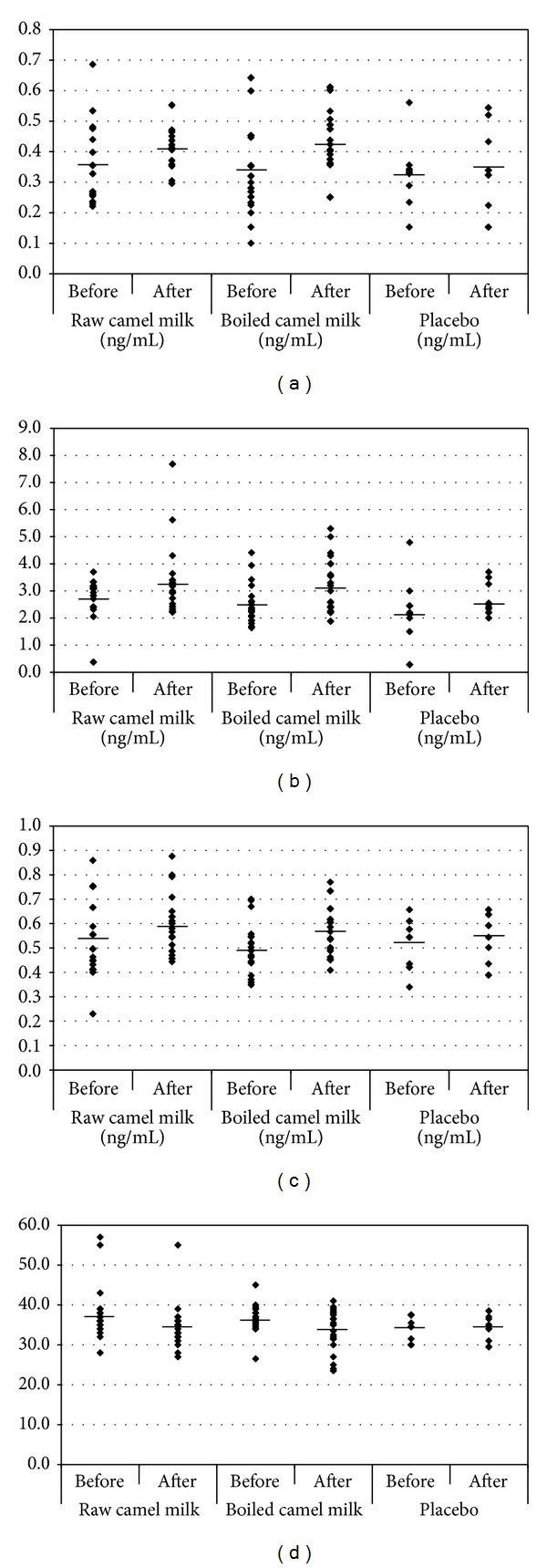
Levels of (a) GSH, (b) SOD, (c) MPO, and (d) CARS in autistic patients before and after treating with the camel milk. The mean value for each group is designated by a line.

Table 1 and Figure 1 show plasma levels of GSH, SOD, and MPO together with CARS of autistic children, before and 2 weeks after camel milk consumption. All measured parameters showed significant changes after camel milk consumption.

Plasma GSH levels were significantly increased in group I and group II (*P* = 0.05, *P* = 0.02, resp.), but not in group III, following 2 weeks of camel milk consumption. In addition, plasma levels of SOD demonstrated no significant differences in group I (*P* = 0.2) and group III (*P* = 0.5). On the other hand, group II demonstrated a highly statistically significant change following 2 weeks of boiled camel milk consumption (*P* = 0.007). Furthermore, there was a significant elevation of MPO in both group I, the raw camel milk (*P* = 0.05), and group II, the boiled camel milk (*P* = 0.02), but not in group III, the placebo group (*P* = 0.2).

**Figure 2:**
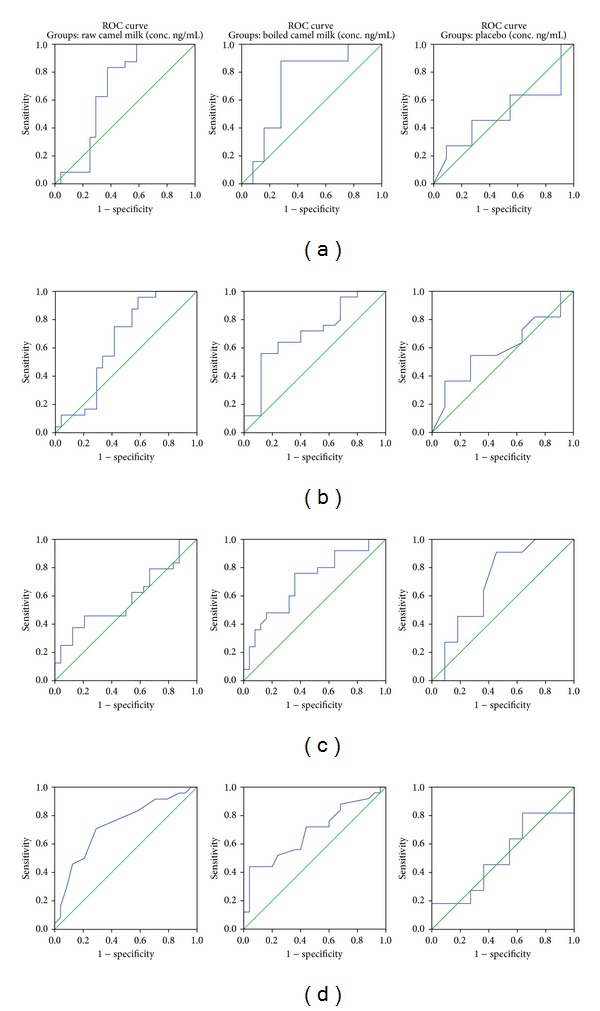
((a)–(d)): ROC curves showing specificity, sensitivity, and area under the curves for (a) GSH, (b) SOD, (c) MPO, and (d) CARS.

Table 2 and Figures 2(a)–2(d) demonstrate ROC analysis of the 4 measured variants. It could be easily noticed that GSH, SOD, MPO, and CARS show higher area under the curve (AUC), % specificity, and sensitivity in groups I and II than in group III. 

## 4. Discussion

The present study aimed at evaluating the effect of camel milk on oxidative stress among subjects with autism spectrum disorders by measuring the levels of antioxidant enzymes: SOD, MPO, and GSH.

Several studies have suggested an increased vulnerability of subjects with ASD to oxidative stress. Oxidative stress and the consequent damage occur when antioxidant defence mechanisms fail to effectively counter endogenous or exogenous sources of reactive oxygen species [38]. Increased oxidative stress might contribute to behavioural aberrations, sleep disorder, and gastrointestinal disturbances in autistic children [39, 40].

Low plasma antioxidant enzymes, GSH-Px [25] and SOD [23], were reported. Low level of antioxidant enzymes indicated increased vulnerability to oxidative stress due to impaired antioxidant defence mechanisms, which lead to harmful effects of free radicals that could have an important role in the aetiology of autism. Moreover, increased oxidative stress in autistic subjects leads to a decrease in the levels of nonenzymatic antioxidants like GSH, vitamin E and C [13], which in turn leads to impairment of metabolic pathways and may contribute to the developmental delays which occur in autism; this could be corrected by micronutrient supplementation [41]. In addition, lower plasma levels of glutathione and cysteine in subjects with ASD were documented [42, 43].

Camel milk has been reported to improve clinical outcomes of ASD [31]. The effect of camel milk consumption on autistic behaviour was documented through significant changes in the Childhood Autism Rating Scale (CARS) scoring results [44], as casein- and gluten-free diet has been reported to improve autistic behavior [31], possibly by reducing excess central opioid effects [45].

Glutathione is one of the most important intracellular antioxidants, responsible for maintaining the reducing intracellular microenvironment that is essential for normal cellular function and viability. It also exerts neuroprotective properties and reduces neuropathy and hence decreases oxidative stress. 

Subjects with ASD were shown to exhibit abnormal plasma levels of metabolites in the pathway of glutathione redox metabolism, due to inefficient detoxification system [12]. The concentration of reduced glutathione (GSH) was found to be significantly decreased compared to control [10, 25], which reflects increased oxidative stress due to the impaired defense mechanisms against ROS. 

The results of the present study show a significant increase in GSH level after camel milk consumption; this could be attributed to the antioxidant nutrients constituents of camel milk. Magnesium is known to reduce oxidative stress and enhance vitamin E and C absorption [44], whereas zinc increases total glutathione, GSHPx, and SOD levels. Moreover, vitamin E has been suggested to enhance glutathione levels [46]. Taken together, high levels of Mg and Zn and vitamin E in camel milk might help to increase glutathione production and enzymes production and hence to decrease the oxidative stress in autistic subjects.

Superoxide dismutase is an antioxidant enzyme that inhibits lipid peroxidation by catalyzing the conversion of superoxide into hydrogen peroxide (H_2_O_2_) and oxygen (O_2_) [13] and acts as a primary defence, as it prevents further generation of free radicals. Insufficient capacity of SOD to metabolize the resulting H_2_O_2_ may lead to toxicity [10].

It was shown that the SOD activity was significantly higher in autistic children compared to control, in response to oxidative stress. The increased activity may be an adaptive response to eliminate superoxide that was excessively produced [10]. In contrast, other studies reported significant decrease of SOD levels in autistic children compared to controls [24, 43], due to the impairment of the defence mechanism against oxidative stress. Low SOD may also contribute to the nutritional status as some of the antioxidant nutrient levels affect the status of the antioxidant enzymes. For example, adequate amounts of superoxide dismutase are produced when the body receives an adequate and balanced intake of copper and zinc. Copper deficiency was reported to reduce the level of superoxide dismutase [23, 46], whereas zinc deficient diet decreases superoxide dismutase, glutathione peroxidase, total glutathione, and vitamin E [47]. Other studies suggested that the low zinc levels have been associated with autism and related to lower SOD levels, due to the lower zinc to copper ratio in autistic children compared to controls [23, 48].

In the present study, SOD level was significantly increased after camel milk consumption; this could be attributed to the high contents of zinc, copper, magnesium, and vitamin E in camel milk.

Myeloperoxidase is a biomarker of oxidative stress that is responsible for microbicidal activity against a wide range of organisms and one of the indicators of inflammation [49]. Elevated superoxide generated from dysfunctional mitochondria promotes the formation of excessive H_2_O_2_, the substrate for MPO-mediated hypochlorous acid synthesis, which is then converted to the inflammatory biomarker, 3-chlorotyrosine (3-CT), in activated immune cells during an inflammatory response [38].

Elevated expression of MPO has previously been demonstrated in chronic neurological disease states, such as Alzheimer's disease [50], Parkinson's disease [51], multiple sclerosis [52], and autism spectrum disorder [53].

It has been demonstrated that autistic children with severe GI disease have low serum levels of MPO, which is directly linked with GI pathology seen in this group [54]. The present study demonstrated a significant increase in the plasma myeloperoxidase level following camel milk consumption, which could be a consequence of increased level of SOD. MPO and SOD work synergistically to protect the cell contents against oxidizing activity by destroying anions and hydrogen peroxide [50]; superoxide dismutase catalyzes the conversion of superoxide radicals to H_2_O_2_, with catalase neutralizing H_2_O_2_ and then myeloperoxidase converting H_2_O_2_ to highly reactive hypochlorous acid [23]. Another possibility might be the improvement of GI problems due to the deprivation of camel milk from beta lactoglobulin and beta casein, the major cause for food allergy and GI disease in autistic subjects [7, 54, 55].

Various studies demonstrated a remarkable improvement of some symptoms in ASD subjects following a gluten- and casein- free diet [34], glutathione supplementation [22], antioxidant supplementation such as vitamin E, C, and selinum [22–24], or magnesium and zinc supplementation [43]. These molecules are essential for glutathione synthesis, antioxidant enzymes activities, antioxidant vitamins absorption, and effective antioxidant defence mechanism and hence they play an important role in decreasing oxidative stress as confirmed in various studies. 

In light of this information, the role of camel milk in decreasing oxidative stress and treatment of ASD could be explained on the basis that it contains high level of antioxidant vitamins C, A, and E and is very rich in antioxidant minerals magnesium and zinc. Antioxidant vitamins are useful in reducing the oxidative stress. Vitamin E and magnesium have been suggested to enhance glutathione biosynthesis. Magnesium deficiency has been associated with the production of reactive oxygen species [46]. On the other hand, zinc is essential for the activity of many enzymes in living organisms such as SOD and GPx. It has been reported that zinc can prevent cell damage through activation of the antioxidant system [47, 56]. Taken together, these nutrients enhance the production of detoxifying molecules, absorption of antioxidant vitamins, and activation of antioxidant enzymes which in turn activate the detoxification system and reduce the exerted oxidative stress. Another possibility is that camel milk can help to combat and treat gastrointestinal problems, which are frequently associated with ASD, due to its inflammation-inhibiting constituents and hypoallergenic properties, in addition to its smaller size antibodies which are similar to human antibodies [7, 37], and thus improve some autistic behaviours. 

The role of the measured parameters in the etiology of autistic features could be also ascertained in this study. The amelioration induced by raw and camel milk on GSH, SOD, and MPO was accompanied by a significant improvement in the behaviour of the autistic children after two weeks of camel milk consumption. CARS was significantly lower after camel milk consumption than before.

Table 2 and Figures 2(a)–2(d) demonstrate that although the four measured parameters did not show very high specificity and sensitivity, GSH and CARS show satisfactory values of both measures. This could help to suggest GSH as a predictive biomarker to follow the potency of camel milk treatment in parallel with the measurement of CARS as a behavioural and cognitional measure. 

In conclusion, our findings suggest that camel milk could play an important role in decreasing oxidative stress by alteration of antioxidant enzymes and nonenzymatic antioxidant molecules levels and improvement of autistic behaviour. A larger scale study considering the period and dosage of camel milk is needed to determine the effect of camel milk on oxidative stress biomarkers and hence the treatment of ASD. In addition, other parameters representing different signalling pathways related to the pathology of autism are recommended. Screening for a predictive marker which might record higher specificity and sensitivity than those of the present study is critically needed. 

## Figures and Tables

**Table 1:** Glutathione, superoxide dismutase, and myeloperoxidase in plasma of autistic children together with CARS before and 2 weeks after camel milk consumption.

	Raw milk (*N* = 24)		Boiled milk (*N* = 25)		Placebo (*N* = 11)	
	Mean ± SEM	*P* value	Mean ± SEM	*P* value	Mean ± SEM	*P* value
Glutathione						
Before	0.37 ± 0.03	0.05	0.34 ± 0.03	0.02	0.36 ± 0.02	0.5
After	0.41 ± 0.01		0.45 ± 0.02		0.35 ± 0.04	
SOD						
Before	0.54 ± 0.03	0.2	0.49 ± 0.02	0.007	0.52 ± 0.03	0.5
After	0.59 ± 0.02		0.57 ± 0.02		0.54 ± 0.03	
MPO						
Before	2.65 ± 0.17	0.05	2.44 ± 0.13	0.02	2.11 ± 0.37	0.2
After	3.22 ± 0.24		3.08 ± 0.19		2.62 ± 0.16	
CARS						
Before	37.63 ± 6.31	0.004	36.82 ± 3.27	0.001	34.18 ± 3.25	0.772
After	34.54 ± 5.19		33.80 ± 4.91		34.41 ± 3.25	

**Table 2:** ROC curve of GSH, SOD, MPO, and CARS of autistic patients before and after treatment with camel milk.

Parameters		Raw camel milk	Boiled Camel milk	Placebo
GSH	Area under the curve	0.677	0.723	0.504
	Best cut-off value	0.357	0.356	0.326
	Sensitivity%	83.3	88.0	45.5
	Specificity%	62.5	72.0	72.7

SOD	Area under the curve	0.642	0.706	0.591
	Best cut-off value	0.453	0.562	0.585
	Sensitivity%	95.8%	56.0%	54.5
	Specificity%	41.7%	88.0%	72.7

MPO	Area under the curve	0.584	0.703	0.702
	Best cut-off value	3.17	2.385	2.180
	Sensitivity%	45.8%	76.0%	90.9%
	Specificity%	79.2%	64.0%	54.4%

CARS	Area under the curve	0.729	0.682	0.512
	Best cut-off value	35.5	33.75	37.25
	Sensitivity%	70.8	44.0	81.8
	Specificity%	70.8	96.0	36.4
